# First study on molecular detection of three major canine tick-borne pathogens in subclinically infected dogs in Chiang Mai, Thailand

**DOI:** 10.14202/vetworld.2022.1121-1128

**Published:** 2022-04-28

**Authors:** Keiichiro Tazawa, Napassorn Poolsawat, Andrew D. Gibson, Luke Gamble, Alasdair King, Panat Anuracpreeda

**Affiliations:** 1 Worldwide Veterinary Service, Cranborne, Dorset BH21 5PZ, United Kingdom; 2 Parasitology Research Laboratory, Institute of Molecular Biosciences, Mahidol University, Salaya, Nakhon Pathom 73170, Thailand; 3 Mission Rabies, Cranborne, Dorset BH21 5PZ, United Kingdom; 4 Merck Animal Health, Madison, New Jersey 07940, United States

**Keywords:** *Anaplasma platys*, canine tick-borne disease, *Ehrlichia canis*, *Hepatozoon canis*, polymerase chain reaction, thin blood smear

## Abstract

**Background and Aim:**

Canine tick-borne pathogens (CTBPs) are an important cause of morbidity in dogs in Thailand. This study aimed to evaluate the occurrence of three CTBPs in clinically normal, owned dogs to understand the risk for the general canine population. We also examined sex, age, tick infestation, and packed cell volume (PCV) of the animals in association with active infection of the CTBPs.

**Materials and Methods:**

A total of 139 dogs were included in the study. Blood samples were collected for thin blood smear, PCV and nested polymerase chain reaction (PCR) assay. Statistical analyses were performed to examine the association between individual factors and CTBP infection status determined by PCR. In addition, sensitivity, specificity, and Cohen’s kappa were calculated to assess the utility of routine blood smear.

**Results:**

The PCR results showed that 31 dogs (22.3%) were infected with at least one of the three pathogens. The occurrence rate for *Ehrlichia canis*, *Anaplasma platys*, and *Hepatozoon canis* was 2.2% (3/139), 18.7% (24/139), and 2.8% (4/139), respectively. There were two cases of coinfection with *A. platys* and *E. canis*. The univariate analyses did not yield any associations between recorded variables and the active infection. Microscopic examination showed good sensitivity and agreement only for *H. canis* (Sn: 75%, 95% confidence interval: 24.9-98.7, k=0.85).

**Conclusion:**

Our findings confirmed the endemicity of the CTBPs in owned canine population in the study site. In-depth epidemiological investigation would be warranted to elucidate environmental risk factors for CTBP infection.

## Introduction

Canine tick-borne diseases (CTBDs) are a significant cause of canine morbidity in Thailand [1]. The prevalent brown dog tick in Thailand, *Rhipicephalus sanguineus*, is a vector for numerous CTBDs, resulting in the increased risk and frequent coinfection with several pathogens and challenges in prevention, diagnosis, and treatment [1,2]. The most widespread canine tick-borne pathogens (CTBPs) in Thailand are *Ehrlichia can*is; *Anaplasma platys*; and *Hepatozoon canis*. *E. can*is is one of the most common CTBPs in Thailand [3-6]. It is a Gram-negative, intracellular bacterium that parasitizes in monocytes and macrophages, and is a cause of canine monocytic ehrlichiosis [7,8]. In addition, *A. platys* is another common bacterial hemoparasite that commonly infects dogs in Thailand, causing canine cyclic thrombocytopenia [3-6,9]. *H. canis* is an apicomplexan parasite characterized by the gamonts found inside leukocytes and is responsible for the disease hepatozoonosis in dogs [4,10]. In general, these CTBDs are transmitted through a blood meal by an infected tick, except for *H. canis*, which is transmitted following ingestion of an infected tick [9,11]. Although the epidemiology of these diseases has not been well elucidated in Thailand, the previous studies suggested the highly heterogeneous spatial occurrence of these CTBDs in dogs throughout the country [3-6,12,13]. Clinical signs for CTBDs are non-specific, and the clinical assessment can be challenging due to underlying diseases, multiple organ systems involved, and variable degrees of parasitemia during infection [10,14,15]. The clinical signs commonly observed in patients with CTBDs include fever, lethargy, anemia, thrombocytopenia, splenomegaly, uveitis, and organ dysfunction, making CTBDs indistinguishable from clinical presentations alone [15-17]. Infected dogs often remain asymptomatic for months or years [18,19] and are, therefore, unlikely to be brought to veterinary attention for testing. In one study conducted in West Indies, only 13% of dogs infected with *E. canis* showed clinical signs, as did an even smaller proportion of dogs infected with *A. platys* and *H. canis* [19]. Coinfection commonly occurs, creating additional challenges in clinical diagnosis [14,20].

Several diagnostic methods have been established for the diagnosis of CTBP infection. Conventional microscopic examination on thin blood smear has been considered unreliable for diagnosing CTBP infection due to markedly lower sensitivity compared with molecular techniques. False positives or negatives can result from the difficulty in morphological identification of parasitic morulae of *E*. *canis* in monocytes or that of *A. platys* in thrombocytes that can resemble artifacts [6,21,22]. While *H. canis* gamonts have a more morphologically recognizable appearance, a previous study showed that the chance of finding them on routine thin blood smear was very low [11]. In addition, detection by blood smear can be affected by the stage of infection due to different magnitudes of parasitemia [22,23]. Serological techniques, such as immunofluorescent antibody test and dot-enzyme-linked immunosorbent assay tests, are more sensitive than microscopic examination of a thin blood smear but cannot distinguish between current and past infections [21,24,25]. Polymerase chain reaction (PCR) has been demonstrated as the most reliable method for detecting active TBP infection in dogs [6,23,24,26]. This molecular technique allows the detection of the CTBPs in the sample even when the antibody titer is below the threshold for serological diagnosis in the early stage of infection [27]. However, the cost and resource requirement for PCR tests are significant limitation for the widespread availability in Thailand [6].

The occurrence of the CTBPs has not been well concluded in CTBD endemic areas, including Thailand, due to pathological characteristics, lack of affordable and sensitive diagnostics for CTBPs, and variable sample populations and locations across studies. Hence, subclinical CTBP infections in owned dogs are not well understood due to the scarcity of epidemiological studies [3,4,28]. Evaluation of the distribution of the CTBDs in healthy domestic dogs could support more effective risk assessment for the diseases and appropriate guidance on tick control for prevention.

To the authors’ best knowledge, this study would be the first to describe the occurrence of CTBP infections only in apparently healthy, owned domestic dogs in Thailand. We believe that this study would also address the necessity to develop more affordable field immunodiagnostic tools as well as consider vaccination schemes.

## Materials and Methods

### Ethical approval and informed consent

All experimental procedures on animals were approved by the Animal Care and Use Committee (IMBMU-ACUC), Institute of Molecular Biosciences, Mahidol University, Thailand. Additionally, consents to collect biological samples were obtained from owners of the animals on admission at the Worldwide Veterinary Service International Training Centre (WVS ITC), Hang Dong District, Chiang Mai Province, Thailand.

### Study period and location

**Figure-1:**
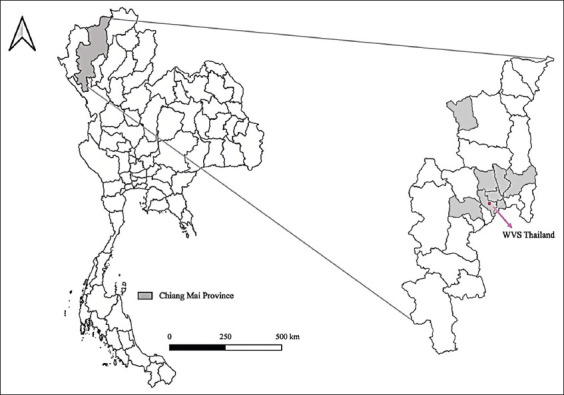
Location of Chiang Mai Province, Worldwide Veterinary Service, Thailand, and sample locations. [Base map source: Humanitarian Data Exchange: https://data.humdata.org/].

The study was conducted from October 2019 to November 2019. Sample collection was conducted at the WVS ITC, Hang Dong District, Chiang Mai Province (Figure-1). Collected samples were analyzed at Parasitology Research Laboratory, Institute of Molecular Biosciences, Mahidol University.

### Sample population

One hundred and thirty-nine mongrel owned dogs were included in this study. These animals were not showing apparent clinical signs such as emaciation, lethargy, diarrhea, and fever. The sex and age of the animals were recorded. The age of the animals was classified into juvenile (aged under 1 year), adult (1-5 years of age), and old (more than 5 years of age). In addition, the presence of tick infestation on the animals was examined and recorded rigorously.

### Collection of blood samples

A sample of 1 ml of peripheral blood from the cephalic vein was collected from each dog and placed into ethylenediaminetetraacetic acid-coated tubes. Blood smear examination and packed cell volume (PCV) evaluation of blood samples were performed immediately following sample collection. The rest of the blood sample was then stored at -20°C for further diagnostic testing using PCR at a later time.

### Microscopic examination

Microscopic examination of thin fresh blood smear was performed for each dog on glass slides immediately after blood collection with the method described by Chawengkirttikul *et al*. [29]. Briefly, smeared slides were fixed with 100% methanol for 1 min and left to air dry before being stained with Giemsa staining (Sigma-Aldrich, Germany) for 40 min. Then, they were observed under a light microscope (Xenon, China) with 100× oil immersion lens for at least 100 fields of well-stained, single-cell layer.

### PCV measurement

A 0.5 μL of blood sample was transferred to a heparinized microcapillary tube (Virtex Medical A/S, Denmark) to evaluate PCV. A tube was then placed into a centrifuge (Portable Centrifuge ZO-1, LW Scientific Inc., USA) and spun at 3357× g for 3 min. Thereafter, PCV levels were measured with a capillary tube reader (LW Scientific Inc., USA) and recorded according to the following criteria: Normal (≥35%), mild (27–34%), moderate (20–26%), and severe (<20%).

### DNA extraction and molecular detection of CTBPs

Frozen samples were transported to Parasitology Research Laboratory (PRL), Institute of Molecular Biosciences, Mahidol University, for the molecular technique. Two hundred fifty microliters of blood samples were used to extract genomic deoxyribonucleic acid (gDNA) of CTBDs (*E. canis*, *A. platys*, and *H. canis*) using Tissue DNA Extraction Kit (Omega Bio-Tex, USA), following the protocol described in the previous studies [30-33]. Positive control samples were obtained from PRL, Institute of Molecular Biosciences, Mahidol University, to exclude false positives due to contamination. DNA extracts were evaluated for their purity, and their concentration was determined by Nanodrop™ 2000 Spectrophotometer (Thermo Scientific™, USA). Nested PCR using two pairs of specific primers to detect the 16s ribosomal ribonucleic acid (rRNA) gene of *E. canis* and *A. platys* was used for amplification, which consists of the first step using universal primers: F 5' AGA-ACG-AAC-GCT-GGC-GGC-AAG-CC 3' and R 5' CGT-ATT-ACC-GCG-GCT-GCT-GGC-A 3`, and the second step using specific primers: PLATYS F 5' TTT-GTC-GTA-GCT-TGC-TAT-G 3' and GA1U R 5' GAG-TTT- GCC-GGG-ACT-TCT-TCT 3' for *A. platys*, and CANIS 5' CAA-TTA-TTT-ATA- GCC-TCT-GGC-TAT-AGG-A 3' and HE3 5' TAT-AGG-TAC-CGT-CAT-TAT-CTT-CCC-TAT 3'for *E. canis*, respectively. Likewise, to detect the 18S rRNA gene of *H. canis*, a pair of specific primers (HCF 5'-ATA-CAT-GAG-CAA-AAT-CTC-AAC-3' and HCR 5'-CTT-ATT-ATT-CCA-TGC-TGC-AG-3') was used. PCR reaction mixtures composed of 50 ng of DNA template, 0.2 mM of the respective primers, 200 mM of each deoxynucleoside triphosphate, standard *Taq* reaction buffer, and 1.25 U Taq DNA polymerase (BioLabs, USA) were prepared, and then, the PCR reactions were carried out in a thermal cycler (Bio-Rad, USA) under the conditions described by Poolsawat *et al*. [28]: 35 cycles of denaturation at 95°C for 45 s, annealing at 60°C and 63°C for the first and second steps of *A. platys*, annealing at 60°C and 53°C for the first and second steps of *E. canis*, annealing at 43°C of *H. canis* for 45 s, and extension at 72°C for 90 s followed by a final extension at 72°C for 5 min. The resultant PCR products were then identified with 1.2% agarose gels stained with FluoroStain™ DNA Fluorescent Staining Dye (Smobio, Taiwan) and observed under ultraviolet transilluminator.

### Statistical analysis

According to the PCR results, binary variables were created to represent the status of infection with the three pathogens and overall CTBP infection. Univariate analyses to assess the association of sex, age, and tick infestation of the animals with the overall CTBP infection status were performed using Pearson’s Chi-squared test. Furthermore, the association between each of the CTBP infection and the PCV level was evaluated by Pearson’s Chi-squared test. The resultant p<0.05 was considered statistically significant. The accuracy of the microscopic examination with a thin blood smear was compared with the PCR assay by calculating sensitivity, specificity, positive predictive value (PPV), and negative predictive value (NPV). Cohen’s kappa coefficient (k) was calculated to see the agreement with the results of PCR. We used the criteria described by Landis and Koch [34] as a reference. All statistical analyses were computed using R version 3.6.1 (R Core Team, 2019) [35].

## Results

### Microscopic examination and PCR assay

Of the 139 canine blood samples examined under a light microscope, the morulae of *A. platys* were found in 4 animals (2.9%), and *H. canis* gamonts were found in 3 animals (2.2%), respectively. *E. canis* morulae could not be observed microscopically in the sample population. On the other hand, the PCR results showed a significantly higher occurrence of CTBP infection in 22% (31/139) of the sampled population infected with at least one of the three pathogens tested, as shown in Table-1. Occurrence scores for *A. platys*, *E. canis*, and *H. canis* were 18.7% (26/139), 2.2% (3/139), and 2.8% (4/139), respectively. Two *E. canis* infected animals were found with coinfection with *A. platys*, totaling 1.3% (2/150) of the sampled population.

**Table 1:** Comparison between PCR assay and microscopic examination.

Pathogens	PCR assay		Blood smear examination	
	
	% positive (95% CI)	Number of dogs	% positive (95% CI)	Number of dogs
*E. canis*	0.7 (0.1-4.0)	1/139	0 (0.0-0.03)	0/139
*A. platys*	18.7 (12.2-27.4)	24/139	2.9 (0.8-7.4)	4/139
*H. canis*	2.8 (0.8-7.4)	4/139	2.2 (0.4-6.3)	3/139
*E. canis+A. platys*	1.4 (0.2-5.1)	2/139	0 (0.0-0.03)	0/139
Overall infection	22.3 (15.2-31.6)	31/139	5.0 (2.9-10.4)	7/139

*E. canis*=*Ehrlichia canis*, *A. platys=Anaplasma platys*, *H. canis*=*Hepatozoon canis*, PCR=Polymerase chain reaction, CI=Confidence interval

### Univariate analyses of demographic characteristics

The results of the univariate analyses regarding the overall CTBPs infection detected by PCR in association with age, sex, and tick infestation are shown in Table-2. As a result, none of the three recorded factors showed a statistically significant association (p<0.05) with the overall CTBP infection.

**Table 2:** Factors associated with CTBP infection detected by PCR assay.

Parameters	Number of dogs examined (%)	% infected dogs (n=31)				% overall CTBP infection detected by PCR (n)	χ^2^ (df)	p-value

		*E. canis* (n)	*A. platys* (n)	*H. canis* (n)	*E. Canis+ A. platys* (n)			
Sex								
Male	38 (27.3)	0 (0/38)	10.5 (4/38)	2.6 (1/38)	0 (0/38)	16.1 (5/38)	2.5 (1)	0.11*
Female	101 (72.7)	1.0 (1/101)	21.8 (20/101)	3.0 (3/101)	2.0 (2/101)	83.9 (26/107)		
Age								
Juvenile (<1 year old)	19 (13.3)	0 (0/19)	21.1 (4/19)	0 (0/19)	0 (0/19)	21.1 (4/19)	0.6 (2)	0.75*
Adult (1-5 years old)	107 (75.3)	0.9 (1/107)	16.7 (18/107)	3.7 (4/107)	1.9 (2/107)	23.4 (25/107)		
Old (>5 years old)	13 (11.4)	0 (0/13)	15.4 (2/13)	0 (0/13)	0 (0/13)	15.4 (2/13)		
Tick infestation								
None observed	34 (24.5)	0 (0/34)	14.7 (5/34)	5.9 (2/34)	0 (0/34)	20.6 (7/34)	0.08 (1)	0.78*
Tick infestation	105 (75.5)	1.0 (1/105)	18.1 (19/105)	1.8 (2/109)	1.9 (2/105)	22.9 (24/105)		

* Chi-squared test, CTBP=Canine tick-borne pathogen, PCR=Polymerase chain reaction, *E. canis*=*Ehrlichia canis*, *A. platys=Anaplasma platys*, *H. canis*=*Hepatozoon canis*, CI=Confidence interval

### PCV measurement and its association with CTBP infection

Table-3 summarizes the results of the PCV measurement. There was evidence of a moderate occurrence of anemia in the sample population. Forty-eight animals (34.5%) had below normal PCV (<35%). In addition, despite being apparently healthy, three animals had severe anemia, but they were free from CTBP infection examined in this study. In clinically healthy animals, there was no association between the PCV levels and active infection with *E. canis*, *A. platys*, and *H. canis*.

**Table 3:** Association between PCV levels and CTBP infection.

Pathogens	Status	PCV levels % dogs (n)				Average PCV (95% CI of the average)	χ^2^ (df)	p-value

		Normal	Mild	Moderate	Severe			
*E. canis*	Positive	0.7 (1/139)	0 (0/139)	0 (0/139)	0 (0/139)	NA	0.53 (3)	0.91*
	Negative	64.8 (90/139)	25.9 (36/139)	6.5 (9/139)	2.2 (3/139)	37.3 (36.1-38.5)	
*A. platys*	Positive	10.8 (15/139)	5.8 (8/139)	0.7 (1/139)	0 (0/139)	37.0 (34.5-39.2)	1.52 (3)	0.68*
	Negative	54.7 (76/139)	20.1 (28/139)	5.8 (8/139)	2.2 (3/139)	37.4 (36.0-37.8)	
*H. canis*	Positive	2.9 (4/139)	0 (0/139)	0 (0/139)	0 (0/139)	42.5 (34.1-50.9)	2.48 (3)	0.48*
	Negative	62.6 (87/139)	25.9 (36/139)	6.5 (9/139)	2.2 (3/139)	37.2 (36.0-38.4)	
*E. canis+ A. platys*	Positive	0 (0/139)	0.7 (1/139)	0.7 (1/139)	0 (0/139)	28.0 (0-78.8)	7.76 (3)	0.051*
	Negative	65.5 (91/139)	25.2 (35/139)	5.8 (8/139)	2.2 (3/139)	37.6 (36.1-38.9)	
Overall CTBP infection	Positive	14.4 (20/139)	6.5 (9/139)	1.4 (2/139)	0 (0/139)	37.2 (35.1-39.4)	1.02 (3)	0.80*
	Negative	51.1 (71/139)	19.4 (27/139)	5.0 (7/139)	2.2 (3/139)	37.4 (35.9-38.8)	
Total (n=139)		65.5 (91/139)	25.9 (36/139)	6.5 (9/139)	2.2 (3/139)	37.7 (36.1-38.5)	NA	

* Chi-squared test; χ^2^=Chi-square, df=Degree of freedom, NA=Not available, CTBP=Canine tick-borne pathogen, PCR=Polymerase chain reaction, *E. canis*=*Ehrlichia canis*, *A. platys=Anaplasma platys*, *H. canis*=*Hepatozoon canis*, PCV=Packed cell volume, CI=Confidence interval

### Diagnostic test comparison between microscopic examination and molecular technique

Microscopic examination of thin blood smears showed low sensitivity for *E. canis*, 0% (95% confidence interval [CI]: 0-63.2%) and *A. platys*, 15.4% (95% CI: 5.4-31.8%) compared with PCR, while there was a moderate sensitivity for *H. canis* microscopically (75%, 95% CI: 24.9-98.7%). Specificities for all three pathogens were close to 100%. PPVs had a wide 95% CI range; 47.8% (95% CI: 10.4-83.0) for *E. canis*, 84.2% (95% CI: 42.7-94.3) for *A. platys*, and 77.7% (95% CI: 31.7-90.0) for *H. canis*, respectively. NPVs, on the other hand, were 97.5% (95% CI: 96.6-98.4), 84.6% (95% CI: 82.7-86.6), and 99.1% (95% CI: 97.5-99.5) for *E. canis, A. platys*, and *H. canis*, respectively. As shown in Table-4, the blood smear for *E. canis* did not agree with PCR (k=0.00) due to no successful microscopic detection of morulae. The agreement for *A. platys* was only fair (k=0.21), while blood smear results for *H. canis* had a very good concordance with PCR to result in a very good agreement (k=0.85).

**Table 4:** Diagnostic capacity of the microscopic examination in comparison with the PCR assay.

Pathogens	Sensitivity % (95% CI)	Specificity % (95% CI)	PPV (95% CI)	NPV (95% CI)	Cohen’s kappa coefficient (k) (95% CI)	Z value	Degree of agreement
*E. canis*	0 (0-63.2)	100 (97.8-100)	47.8 (10.4-83.0)	97.5 (96.6-98.4)	0 (0-0)	NA	No agreement
*A. platys*	15.4 (5.4-31.8)	100 (97.4-100)	84.2 (42.7-94.3)	84.6 (82.7-86.6)	0.23 (0.04-0.42)	2.38	Fair
*H. canis*	75 (24.9-98.7)	100 (97.8-100)	77.7 (31.7-90.0)	99.1 (97.5-99.5)	0.85 (0.57-1.00)	5.91	Very good

PPV=Positive predictive value, NPV=Negative predictive value, NA=Not available, PCR=Polymerase chain reaction, CI=Confidence interval

## Discussion

We aimed to present the evidence of the persistent transmission of the CTBDs by investigating the occurrence in an apparently healthy, owned canine population in Chiang Mai Province and demonstrate the significant superiority of PCR compared to the conventional microscopic examination using thin blood smear in terms of CTBD diagnostic capability. The present study showed an overall moderate occurrence of CTBDs in the sample population and the necessity of the molecular technique for accurate diagnosis.

According to the previous studies [4,6,13,36] carried out in Thailand, the occurrence rates varied between different hemoparasites. For instance, a study conducted in Buriram Province in Thailand had an occurrence of *H. canis* and *A. platys* (4.1% and 31.6%, respectively) but had a much higher *E. canis* occurrence (36.7%) in the stray canine population [6]. Likewise, a study focused on the stray dog population in Mahasarakham Province [4] showed a higher occurrence of *E. canis* (43.1%) and *H. canis* (12.3%), whereas that of *A. platys* was similar (29.2%) to this study. However, another study conducted in Songkhla Province [36] showed a low occurrence of *E. canis* (1.1%) and *A. platys* (3.3%) in the stray canine population. On the other hand, a study focused on domestic dogs in Khon Kaen Province [13] showed a very similar *E. canis* occurrence (3.0%) to this study. It is important to note that these variations in the occurrence of CTBPs are attributed to differences in the methods employed in these studies such as subject recruitment and study sites, as well as the pathological significance of these diseases. For example, animals infected with *E. canis* would be more likely to present with clinical signs compared with *A. platys* and *H. canis* [4,5,19], resulting in a lower occurrence in this sample population due to the subject selection. These subclinically infected animals are considered to be the natural reservoir of CTBDs [19,37].

Our results for the statistical analyses demonstrated that sex, age, and tick infestation of the animals did not show a correlation with active CTBP infection. Although visible tick infestation was common in the sample population (75.5%), it did not necessarily indicate active CTBP infection, which conforms with the previous study conducted in Thailand [4]. However, a study in Brazil showed a strong association between tick infestation and canine ehrlichiosis in apparently healthy dogs [38]. This disagreement was possibly due to the low occurrence of the CTBPs in the tick population, failure of transferring pathogen from ticks to healthy canine population, or significantly low magnitude of parasitemia [39]. Regardless, this high proportion of dogs with tick infestation suggests a lack of effective ectoparasitic prevention and the dogs’ frequent access to the outside environment as commonly practiced in Thailand [40]. One extensive epidemiological survey showed certain environmental factors such as roaming status, hygiene condition of the household, rural locations of the household, and lack of access to veterinary treatment and antiparasitic treatment showed a significant association with canine ehrlichiosis in healthy animals [39]. A study conducted in Turkey [41] also presented a higher occurrence of CTBDs in stray dogs, suggesting that regular access to the environment could increase the chance of infection. In the case of Thailand, the same would be inferred for owned canine population due to the common ownership practice of allowing dogs to roam freely. Hence, another implication of this study is that the circulation of the CTBPs in the study site is maintained due to the combination of difficulty in definitive diagnosis, variable clinical presentation, and ownership practice. Thus, community-based epidemiological investigation to analyze the risk of CTBDs would identify environmental factors contributing to the higher chance of CTBP exposure, which was found particularly beneficial in understanding disease epidemiology [38,42,43].

Likewise, our investigation of PCV levels in apparently healthy dogs did not yield a significant association with CTBP infection. The previous studies that examined the relevance of PCV levels with *E*. *canis* infection showed that dogs infected with *E. canis* had a significantly lower PCV in apparently healthy dogs [5] and stray dogs [4]. However, this significant reduction in PCV in infected animals was not observed in the present study. In support of this study outcome, an experimental study conducted by Waner *et al*. [37] described that dogs purposely infected with *E. canis* had no significant anemia during the subclinical phase of the infection and the decline of PCV levels was inconsistent. With regard to *A. platys* and *H. canis*, our results showed the chances of these infections manifested as abnormal PCV appeared low, which is in agreement with the previous studies [5,11,14,19]. Coinfection of CTBDs in association with laboratory parameters has been documented in several studies, and the presentation is as variable as a single infection [4,5,15,19,44]. Coinfection of *E. canis* and *A. platys* showed a negative correlation with PCV in a study conducted by Piratae *et al*. [5]. However, there are insufficient data presenting coinfection of CTBPs in terms of clinicopathological parameters, largely due to lower occurrence compared with a single infection.

With respect to diagnostic technique, our findings indicated that the sensitivity of microscopic examination was far lower for *E. canis* and *A. platys* when compared with the molecular techniques, which conforms with the previous studies [6,14,21,36,45]. Particularly for *E. canis*, no morulae were found during the microscopic examination. This failure would be likely due to a low degree of parasitemia [14] and was unremarkable given the sample population likely in the subclinical phase of *E. canis* infection. It is also important to note that PCR tests using peripheral blood samples can fail to detect *E. canis* during the subclinical stage of infection due to the marginalization of the pathogen in certain organs, such as the spleen, liver, and bone marrow [46]. Therefore, our results may have underestimated the occurrence of *E. canis* in the sample population. Regarding *A. platys*, the sensitivity of the microscopic examination was poor, and the degree of agreement with PCR was fair. The morphological recognition of the inclusion body of *A. platys* is considered strenuous as it is frequently mistaken for intracellular artifacts formed during platelet activation [21]. Meanwhile, the sensitivity of thin blood smear for *H. canis* was high compared with the previous studies [6,11]. In addition, the morphological characteristics of *H. canis* made it easier to identify the intracellular gamonts.

Several limitations should be considered when interpreting the results of the present study. First, another CTBP of significance in the area, *Babesia* spp., was not tested in the sample population. Therefore, the occurrence rate of overall CTBP infection might be higher if *Babesia* spp. was tested in the population. Second, the study subject was apparently healthy, owned animals, which is a biased sample population. Finally, although no studied characteristics were found significant in association with active CTBP infection in this study population, a more in-depth epidemiological investigation would be of major benefit to elucidate underlying animal and environmental factors concerning CTBP infection.

## Conclusion

This study demonstrated a moderate occurrence of the three CTBDs in owned, clinically normal dogs in Chiang Mai Province, confirming the endemicity in the study site. *A. platys* was most common, with a lower occurrence of *E. canis* and *H. canis* in the sample population. These subclinically infected dogs are considered to play a role in maintaining the natural burden of the diseases. Along with a high occurrence of tick infestation, the findings from this study would be best used to inform dog owners of the importance of regular ectoparasitic control. Although this study showed a moderate sensitivity for microscopic detection of *H. canis*, there were only a small number of positive samples in the study population. The overall superiority of the molecular technique in CTBP detection was considerable compared with the microscopic examination. Given that access to and resources for molecular diagnosis are a significant limitation to the definitive diagnosis of CTBDs in Thailand, the development of a more affordable, sensitive field test would expedite treatment at an earlier stage of infection.

## Authors’ Contributions

KT: Conceptualization, methodology, data analysis, validation, and drafting of the original manuscript. NP: Laboratory experimentation, revision of the manuscript, and resources. ADG: Data curation, supervision, and manuscript revision. LG and AK: Resources and funding acquisition. PA: Supervision, resources, manuscript revision, funding acquisition, and validation. All authors read and approved the final manuscript.
